# Analysis of the blood bacterial composition of patients with acute coronary syndrome and chronic coronary syndrome

**DOI:** 10.3389/fcimb.2022.943808

**Published:** 2022-10-04

**Authors:** Ikram Khan, Imran Khan, Muhammad Usman, Zhou Jianye, Zhang Xiao Wei, Xie Ping, Li Zhiqiang, An Lizhe

**Affiliations:** ^1^ Department of Microbiology, School of Life Sciences, Lanzhou University, Lanzhou, China; ^2^ School of Stomatology, Northwest Minzu University, Lanzhou, China; ^3^ Department of Microbiology, Khyber Medical University Peshawar, Peshawar, Pakistan; ^4^ State Key Laboratory of Grassland Agro-ecosystem, Key Laboratory of Grassland Livestock Industry Innovation, Ministry of Agriculture and Ruler Affairs, College of Pastoral Agriculture Sciences and Technology, Lanzhou University, Lanzhou, China; ^5^ Department of Cardiology, Lanzhou University Second Hospital, Lanzhou, China; ^6^ Department of Cardiology, Gansu Provincial Hospital, Lanzhou, China

**Keywords:** bacteria, blood microbiota, acute coronary syndrome, chronic coronary syndrome, novaSeq

## Abstract

Emerging evidence revealed that the blood microbiota plays a role in several non-communicable diseases, including cardiovascular disease. However, the role of circulating microbes in atherosclerosis remains understudied. To test this hypothesis, we performed this study to investigate the microbial profile in the blood of Chines atherosclerosis volunteers. A total of seventy Acute Coronary Syndrome patients, seventy Chronic Coronary Syndrome patients, and seventy healthy individuals were examined using high-throughput Illumina Novaseq targeting the V3-V4 regions of the 16S rRNA gene. The relationship between atherosclerosis and blood microbiome, clinical variables, and their functional pathways were also investigated. Our study observed significantly higher alpha diversity indices (Chao1, *p* = 0.001, and Shannon, *p* = 0.004) in the acute coronary syndrome group compared with chronic coronary syndrome and healthy group, although a significantly lower alpha diversity was observed in the chronic coronary syndrome compared to acute coronary syndrome and healthy group. Beta diversity based on principal coordinate analysis demonstrated a major separation among the three groups. In addition, using linear discriminant analysis, a significant distinct taxon such as Actinobacteria _ phylum, and *Staphylococcus_* genus in the healthy group; Firmicutes_ phylum, and *Lactobacillus*_ genus in the chronic coronary syndrome group, and Proteobacteria and Acidobacteriota _ phyla in acute coronary syndrome group were observed among three groups. Clusters of Orthologous Genes grouped and Kyoto Encyclopedia of Genes and Genomes pathways suggested a significant variation among all groups (*p < 0.05*). The blood microbiota analysis provides potential biomarkers for the detection of coronary syndromes in this population.

## Introduction

Coronary artery disease is a major cause of morbidity and mortality worldwide. Chronic coronary syndrome is defined by the different evolutionary stages of Coronary artery disease (Lyu et al., 2021), but does not include cases with clinical manifestations dominated by the acute coronary syndrome. It emphasizes that the stability of non-acute coronary heart disease is only relative, and there is a risk of progression to Acute coronary syndrome at any time, which leads to cardiovascular events (Steg et al., 2007). Acute coronary syndrome is also a common subcategory of cardiovascular disease and has led to increased mortality globally (Zheng et al., 2021). Acute coronary syndrome is a set of signs and symptoms due to acutely decreased blood flow in the coronary arteries, and the exact mechanism underlying its pathogenesis remains to be fully elucidated (Dai et al., 2020). According to 2016 statistics from the “American College of Cardiovascular Diseases”, the incidence of chronic coronary syndrome is about twice that of myocardial infarction and is expected to affect 18% of adults by 2030 (Mozaffarian et al., 2016). Coronary artery diseases are associated with increased bacterial translocation in the gastrointestinal as well as bloodstream infection. The intestinal microbiota and its metabolites have been implicated in the development of atherosclerosis in several investigations (Amar et al., 2019). However, the role of circulating microorganisms in atherosclerosis remains unstudied. From this perspective, developing new atherosclerosis biomarkers is crucial to developing early targets for disease detection and treatment.

Several studies have documented an association between chronic infections and the risk of cardiovascular disease (Armingohar et al., 2014; Lund Håheim, 2014; Lawrence et al., 2022). The circulation is a closed system, and the blood in healthy individuals was earlier believed to represent a sterile environment, which is the basis for safe blood transfusions (Damgaard et al., 2015). However, a previous study reported the presence of bacterial DNA in healthy human blood (Nikkari et al., 2001), and another study defined a healthy human blood microbiome in 2008 (Moriyama et al., 2008). Furthermore, the blood microbiome’s potential interactions with other human microbiomes were discussed in a review of blood microbiome studies (Castillo et al., 2019). All the biological processes by which bacteria may influence circulation are not clarified, but the main processes are atherosclerosis and thrombosis. Atherosclerosis is well known as a low-grade, chronic inflammation of the arterial wall, and is an important factor in the development of several diagnoses of cardiovascular disease. For many years bacteria have been suspected to be part of the pathogenesis of this group of diseases, as whole bacteria, fragments, and their DNA have been identified in blood from cardiovascular disease patients (Koren et al., 2011; Amar et al., 2013; Armingohar et al., 2014; Dinakaran et al., 2014; Lund Håheim, 2014). Several other key processes may be responsible for atherosclerosis; among them accumulation of monocyte/macrophage lineage cells within the lipid-rich subendothelial space of the affected artery where bacterial lipopolysaccharide (LPS) also participates in the formation of macrophage-derived foam cells (Tabas and Lichtman, 2017; An et al., 2018). The accumulation of lipid bodies in the foam cells is affected by both the nutrition and gut microbiota. Bacteria are also known to form thrombi and emboli in interaction with platelets, and this is also seen as a complication of advanced lesions of atherosclerosis (Armingohar et al., 2014; Lund Håheim, 2014; Lawrence et al., 2022).

Gut bacteria may act as key “metabolic filters” of the diet. They can convert common nutrients to metabolites, such as specific microbial-associated metabolites, such as trimethylamine-N-oxide and short-chain fatty acids. These components including secondary bile acid have also been shown to affect the progression of cardiovascular disease. Furthermore, gastrointestinal infection or autoimmune diseases such as gluten intolerance may contribute to the leakage of bacteria into the circulation and promote atherosclerosis (Kozarov, 2012; Rodrigues et al., 2012). Bacterial DNA identified in blood or serum may represent live bacteria, cultivable or uncultivable bacterial species as well as dormant (non-dividing) bacteria (Potgieter et al., 2015). Live bacteria and their membrane vesicles may enter the bloodstream *via* leaking epithelial junctions or mucosal disruptions (Delzenne et al., 2011). The lung microbiome has also been defined and shown how it may change during disease (Dickson and Huffnagle, 2015). The constitution of the respiratory microbiome is determined by three factors: microbial immigration, elimination, and the relative reproduction rates of its members. Those factors may also influence the blood microbiome during cardiovascular disease. In patients with untreated advanced periodontal disease, chewing and tooth brushing may also result in chronic bacteremia by the migration of bacteria from the subgingival biofilm through the junctional epithelium and into blood vessels in the connective tissue (Könönen et al., 2019).

Based on previous studies, a possible alteration in the microbiota of the blood of patients with Acute coronary syndrome and Chronic coronary syndrome has been hypothesized. By sequencing 16S rRNA gene sequences, this study was able to characterize and compare the blood bacterial profiles of patients with acute coronary syndrome and chronic coronary syndrome with those of healthy controls. Analyzing the blood bacterial community was designed to determine whether a particular microbiota is associated with a particular disease. Additionally, we examined the relationship between the microbiota in the blood of acute coronary syndrome and chronic coronary syndrome patients and their clinical characteristics. Blood microbiota changes could provide insight into the origin, causes, and implications of atherosclerosis.

## Materials and methods

### Ethics statement

This study was approved by the Research Ethics Committee of Northwest Minzu University and the Gansu Provincial People’s Hospital (Approval No: XBMZ-YX-2021008), both in Lanzhou, China. All participants were informed of the study’s purpose and provided informed consent following the Declaration of Helsinki.

### Study subjects

In the current study, 210 volunteers were enrolled, with 70 patients undergoing acute coronary syndrome and 70 patients with chronic coronary syndrome admitted to the Department of Cardiology, Gansu Public Provincial Hospital, and the physical examination center of Lanzhou University Second Hospital in Gansu Province, China, was used to select 70 healthy individuals. Each participant’s demographic, clinical, and biological information was documented. The following were the patient group’s inclusion criteria: (i) ECG requirements include ST-segment elevation or new left bundle-branch block in two or more standard leads, or at least 2 mm in two or more consecutive precordial leads; and (ii) angiographically verified coronary thrombi. Chronic viral infections (including hepatitis C, HIV, and herpes simplex type 2), chronic inflammatory bowel disease, renal failure, and pregnancy were also removed from both groups. Within 15 days after the acute ischemia event, the majority of ACS (50/70; 71%) and CCS (59/70; 84%) patients were included. Healthy people without a history of cardiovascular diseases or active infection symptoms were considered. All volunteers were ethnic Chinese.

### Samples collection, DNA extraction, amplicon sequencing, and statistical analysis

Clinically certified team members drew blood samples in Vacutainer EDTA Blood Collection Tubes. Reagents and materials were disinfected and wore lab clothes, masks, and disposable gloves to avoid contamination of foreign DNA. For each volunteer 3 ml blood sample was drawn in the morning following overnight fasting conditions and stored at -80°C.

DNA extraction and implication of V3-V4 regions of 16Sr RNA gene were amplified by using 338F: 5’-ACTCCTACGGGAGGCAGCA-3’ and 806R: 5’-GGACTACHVGGGTWTCTAAT-3’ universal primer set by following our previous study protocols (Khan et al., 2022b). Followed by Amplicons sequencing using Illumina Novaseq 6000. Graph Pad Prism (V=8.0) was used to do all statistical analyses on obtained data. The mean and standard deviation were used to present all the data. One-way ANOVA and Tukey’s multiple comparisons *post-hoc* test were used for clinical parameters such as, High-density lipoprotein, Low-density lipoprotein, Tryglyceride, and Body mass index. A negative control sample of sterile water was also utilized to purify DNA libraries (**Supplementary Figure S1**). The importance of several factors was determined using a *p*-value less than 0.05.

## Results

### Clinical characteristics of the studied groups

We recruited 70 ACS patients, 70 CCS patients, and 70 healthy subjects. (**Table 1**) summarizes the clinical indexes such as age, gender, and body mass index that were compared between the three groups. The patients with acute and chronic coronary syndromes were older than healthy individuals (61.8 ± 12.1; 62.58 ± 11.16; 41.2 ± 10.1). Based on a one-way ANOVA test, we found that acute coronary syndrome, chronic coronary syndrome, and healthy groups differed significantly in age, height, body mass index, systolic blood pressure, diastolic blood pressure, high-density lipoprotein, fasting blood glucose, total cholesterol, hypertension, diabetes, and smoking. In contrast, there was no significant difference between the three groups in terms of gender, weight, triglycerides, and low-density lipoprotein (**Table 2**).

**Table 1:** Demographic and clinical characteristics of subject groups.

Characteristics	Healthy (n = 70)	ACS (n = 70)	CCS (n = 70)
Female (n, %)	26 (37.14%)	15 (21.42%)	19 (27.14%)
Male (n, %)	44 (62.86%)	55 (78.58%)	51 (72.86%)
Age (year)	41.2 ± 10.1	61.8 ± 12.1	62.58 ± 11.16
BMI (kg/m^2^)	24.37 ± 3.80	23.75 ± 2.94	25.97 ± 3.05
Current smoker (n, %)	0	13 (18.57%)	21 (30%)
Hypertension (n, %)	0	19 (27.14%)	23 (32.85%)
Diabetes (n, %)	0	20 (28.57%)	21 (30%)
Systolic blood pressure	116.92 ± 12.84	131.58 ± 20.08	127.6 ± 20.91
Diastolic blood pressure	72.74 ± 9.82	78.42 ± 11.94	74.94 ± 15.22
LDL (mmol/L)	2.40 ± 0.71	2.49 ± 0.85	2.36 ± 1.34
HDL (mmol/L)	1.12 ± 0.30	0.97 ± 0.19	1.06 ± 0.25
Triglycerides (mmol/L)	1.64 ± 1.06	1.71 ± 1.17	1.71 ± 0.89
Fasting blood glucose (mmol/L)	5.17 ± 0.57	7.55 ± 3.87	7.11 ± 2.68
Total cholesterol (mmol/L)	4.14 ± 0.73	3.66 ± 136	3.70 ± 0.98

Data are represented as a percentage, mean and standard deviation. ACS, Acute coronary syndrome; CCS, Chronic coronary syndrome; BMI, Body mass index; LDL, Low-density lipoprotein; HDL, High-density lipoprotein; mmol/L, Millimoles per liter; n, Number.

**Table 2:** P-values across Acute coronary syndrome, Chronic coronary syndrome, and Healthy groups.

Characteristics	ACS Vs CCS	ACS VS Healthy	CCS VS Healthy
Age	0.899	0.000^*^	0.000^*^
Weight	0.667	0.297	0.801
Height	0.000^*^	0.757	0.000^*^
Body mass index	0.000^*^	0.511	0.012
Systolic blood pressure	0.404	0.000^*^	0.002^*^
Diastolic blood pressure	0.229	0.021^*^	0.553
Low-density lipoprotein	0.719	0.847	0.972
High-density lipoprotein	0.090	0.002^*^	0.352
Triglycerides	1.000	0.930	0.918
Fasting blood glucose	0.604	0.000^*^	0.000^*^
Total cholesterol	0.962	0.022^*^	0.044^*^
Gender	0.612	0.063	0.384
Diabetes mellitus	0.000^*^	0.000^*^	0.000^*^
Smoking	0.132	0.005^*^	0.000^*^
Hypertension	0.642	0.000^*^	0.000^*^

p-values were shown among the three groups by comparing their clinical characteristics using the One-way ANOVA test. The significant difference was mentioned in bold font with a star. ACS vs CCS, Acute coronary syndrome group vs Chronic coronary syndrome; ACS vs Healthy, Acute coronary syndrome group vs Healthy; CCS vs Healthy, Chronic coronary syndrome group vs Healthy.

### Filtering and sequencing of DNA

**Figure 1:**
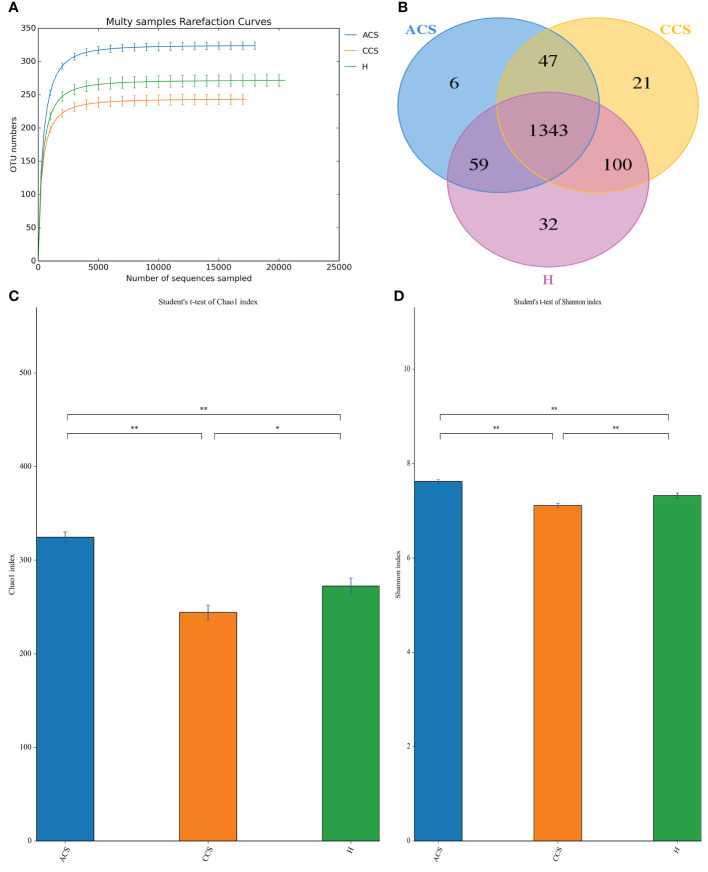
OTUs curve and Venn diagram of ACS, CCS, and H groups. **(A)** Curves illustrate species accumulation between the number of samples and the estimated richness. In each group, the predicted OTU richness was close to saturation. **(B)** A Venn diagram displaying the overlaps among ACS, CCS, and H groups. **(C)** Represent the Chao1 index between three groups. **(D)** Shows the Shannon index between three groups. OTUs, Operational taxonomic units; ACS, Acute coronary syndrome; CCS, Chronic coronary syndrome; H, healthy. "*" represent significance "**" shows strong significane.

After sequencing 210 blood samples, a total of 16,809,565 paired-end reads were generated. Upon that, 16,786,147 clean reads were obtained after paired-end reads quality control and assembly. Each sample generated a minimum of 79,267 clean readings and an average of 79,934 clean reads. Finally, a total of 1,608 OTUs were obtained among all three groups. According to the species accumulation curve, the predicted OTUs richness has already exceeded saturation at this sequencing depth, suggesting that most of the variety has been observed (**Figure 1A**). A Venn diagram revealed that 1,343 of the total 1,608 OTUs were shared throughout the three groups, with 6 OTUs being unique to acute coronary syndrome, 21 to chronic coronary syndrome, and 32 to healthy group (**Figure 1B**).

### Richness and diversity of the blood microbiota

The alpha diversity of blood microbiota among acute coronary syndrome, chronic coronary syndrome, and healthy was measured using the Chao1, observed species, ACE, Coverage, Shannon, and Simpson indices (**Table 3**). A significantly higher Alpha diversity Chao1 index (**Figure 1C**), and Shannon index (**Figure 1D**) were observed in acute coronary syndrome compared to chronic coronary syndrome, and healthy groups although, the chronic coronary syndrome group was significantly lower alpha diversity compared to acute coronary syndrome and healthy groups. A higher coverage indicates that a species in the sample is more likely to be detected. This value is used to evaluate whether the sequencing data is adequate to present the real situation of the microbial community in the sample.

**Table 3:** The comparison of blood microbiota alpha diversity between each group.

Indices	Healthy individuals	Acute coronary syndrome	Chronic coronary syndrome	*p*-value
Shannon	7.3217 ± 0.0564	7.6203 ± 0.0373	7.1087 ± 0.0475	0.004**
Simpson	0.9896 ± 0.0008	0.9916 ± 0.0004	0.9874 ± 0.0007	0.02*
ACE	272.6048 ± 8.7126	324.5061 ± 5.6822	244.8112 ± 7.9086	0.01*
Chao1	272.3857 ± 8.7255	324.4619 ± 5.6876	244.1786 ± 7.9358	0.01*
PD	26.0812 ± 0.7942	23.0523 ± 0.5254	23.4157 ± 0.8261	0.215

Alpha diversity showed significant differences among groups represented with (*) and (**). A single asterisk (*) indicates significance, and the double-asterisk (**) shows strong significance. ACE, Abundance-based coverage estimators; PD, Phylogenetic diversity.

**Figure 2:**
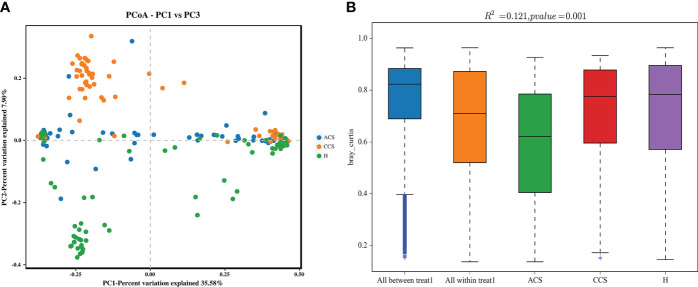
**(A)** Principal coordinate analysis (PCoA) of the overall composition of the genera communities among both groups. **(B)** PERMANOVA analysis indicates variation in blood bacterial species between HC and MI groups.

We performed a beta diversity analysis to examine and contrast the differences and similarities in microbial population structure between the groups. (**Figure 2A**) shows that the three groups had different microbial compositions (PC1 = 35.58 percent and PC2 = 7.90 percent) using Bray-Curtis-based principal coordinate analysis (PCoA). PERMANOVA indicated that the blood bacterial communities among the three groups clustered significantly separated from each other (*R^2^* = 0.191, *p* = 0.001), as depicted in (**Figure 2B**). Finally, the investigations demonstrated that the microbial composition of the acute coronary syndrome and chronic coronary syndrome groups differed from that of the healthy group, implying that the microbial ecology of the patient’s blood is shifting.

### Taxonomic comparison of blood microbiota at the phylum and genus levels

**Figure 3:**
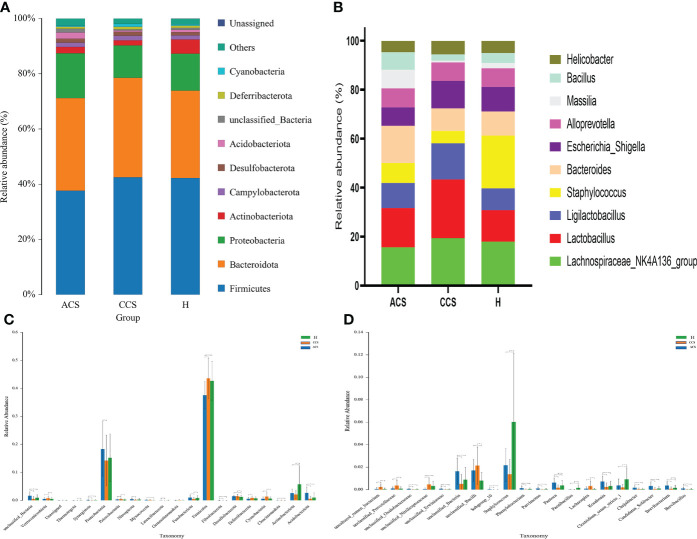
Composition of blood microbiota among acute coronary syndrome, chronic coronary syndrome, and healthy groups. **(A)** The data shown represent the top 10 most abundant phyla among acute coronary syndrome, chronic coronary syndrome, and healthy groups. **(B)** The top ten genera among acute coronary syndrome, chronic coronary syndrome, and healthy groups are shown by the data. **(C)** The ANOVA test indicates a significant change in the proportion among acute coronary syndrome, chronic coronary syndrome, and healthy groups at the phylum level. **(D)** A significant change in the proportion among acute coronary syndrome, chronic coronary syndrome, and healthy groups at the genus level.

At the phylum level, the blood microbiota of the acute coronary syndrome, chronic coronary syndrome, and healthy groups was dominated by Firmicutes (39%, 45%, 43%) Bacteroidetes (31%, 32%, 29%), and Proteobacteria (19%, 15%, 15%), (**Supplementary Table S1** **).** The relative abundance of the top 10 blood bacterial phyla between the three groups, see (**Figure 3A**), accounted for up to 95% of the relative abundance on average. Bacterial genera *Lachnospiraceae NK4A136 group* (16%, 19%, 18%)*, Lactobacillus* (16%, 24%, 13%), and *Ligilactobacillus* (10%, 15%, 9%) (**Supplementary Table S2** **),** the high abundance genera were observed between three groups (**Figure 3B**). The phylum and genus level microbial composition of all blood samples is shown in (**Supplementary Figures S2** and **S3**).

### Acute coronary syndrome vs. chronic coronary syndrome

First, we examined how microbial communities differed between diseased groups. Proteobacteria (19% vs 9%) were overrepresented, while Firmicutes and Bacteroidota (44% vs 38% and 32% vs 31%) were underrepresented in ACS compared to the CCS group. The significant abundance of phylum Acidobacteriota (0.026 ± 0.0194 vs 0.005 ± 0.009; *p* < 0.001), Followed by Actinobacteriota (0.025 ± 0.014 vs 0.021 ± 0.015; *p* > = 0.1), Cloacimonadota (0.0475 ± 0.0748 vs 0.0229 ± 0.0989; *p* > = 0.1), Fibrobacterota (0.028 ± 0.048 vs 0.0009 ± 0.0003; *p* < 0.01), Fusobacteriota (0.009 ± 0.007; 0.005 ± 0.005; *p* < 0.001), Latescibacterota (0.0005 ± 0.001 vs 0.00006 ± 0.00043; *p* < 0.01), Myxococcota (0.0016 ± 0.002 vs 0.0010 ± 0.0017; *p* < 0.1), Nitrospirota (0.0037 ± 0.0031 vs 0.0015 ± 0.0027; *p* < 0.001), Proteobacteria (0.1827 ± 0.0978 vs 0.1422 ± 0.0911; *p* < 0.05), Synergistota (0.0014 ± 0.0014 vs 0.0003 ± 0.0009; *p* < 0.001), and Thermotogota (0.0002 ± 0.0003 vs 0.00001 ± 0.00008; *p* < 0.1) were significantly increased, although Cyanobacteria (0.005 ± 0.007 vs 0.013 ± 0.013; *p* < 0.001), Deferribacterota (0.005 ± 0.005 vs 0.008 ± 0.005; *p* < 0.01), Desulfobacterota (0.014 ± 0.003 vs 0.014 ± 0.006; *p* > = 0.1), Firmicutes (0.375 ± 0.049 vs 0.435 ± 0.070; *p* < 0.001), Gemmatimonadota (0.0004 ± 0.0006 vs 0.0008 ± 0.0020), Patescibacteria (0.0028 ± 0.0024 vs 0.0042 ± 0.0034; *p* < 0.02), Verrucomicrobiota (0.005 ± 0.004 vs 0.007 ± 0.007; *p* < 0.05) were observed significantly decreased in acute coronary syndrome group compared to chronic coronary syndrome (**Figure 3C**).

At the genus level, we observed that *Lachnospiraceae_NK4A136_group* (15% vs19%), and *Lactobacillus* (15% vs 23%) were found to decrease in the acute coronary syndrome group than chronic coronary syndrome. In addition, the considerable increase proportion of *Brevibacillus* (0.0010 ± 0.0010 vs 0.0000 ± 0.0003; *p* < 0.001), followed by *Brevibacterium* (0.0036 ± 0.0025 vs 0.0007 ± 0.0016; *p* < 0.001), *Candidatus_Solibacter* (0.0031 ± 0.0031 vs 0.0004 ± 0.0015; *p* < 0.001), *Chujaibacter* (0.0017 ± 0.0015 vs 0.0003 ± 0.0012; *p* < 0.001), *Clostridium_sensu_stricto_1* (0.0036 ± 0.0057 vs 0.0019 ± 0.0026; *p* > = 0.1), *Kosakonia* (0.0071 ± 0.0048 vs 0.0023 ± 0.0036; *p* < 0.001), *Paenibacillus* (0.000086 ± 0.000358 vs 0.000008 ± 0.00007; *p* > = 0.1) *Pantoea* (0.0061 ± 0.0040 vs 0.0016 ± 0.0026; *p* < 0.001), *Parvimonas* (0.0010 ± 0.0009 vs 0.0000 ± 0.0003; *p* < 0.001), *Phenylobacterium* (0.0012 ± 0.0015 vs 0.0001 ± 0.0005; *p* < 0.001), *Staphylococcus* (0.0217 ± 0.014 vs 0.0136 ± 0.013; *p* > = 0.1), while, *Lachnospira* (0.0008 ± 0.0017 vs 0.0030 ± 0.0045; *p* < 0.001) were observed decreased in acute coronary syndrome group than chronic coronary syndrome (**Figure 3D**).

### Acute coronary syndrome vs. healthy

Bacteroidota and Proteobacteria (31.2% vs 28% and 19% vs 15%) were the most predominant phylum in the acute coronary syndrome group, while Firmicutes (43% vs 38%) were observed enriched in healthy. Further, The proportion of Acidobacteriota was significantly greater (0.0264 ± 0.0194 vs 0.0100 ± 0.0165; *p* < 0.001), Cloacimonadota (0.000475 ± 0.000748 vs 0.000081 ± 0.000247; *p* > 0.01), Desulfobacterota (0.0144 ± 0.0036 vs 0.0119 ± 0.0053; *p* < 0.02), Fibrobacterota (0.0002 ± 0.0004 vs 0.000088 ± 0.000286; *p* < 0.01), Fusobacteriota (0.0098 ± 0.0070 vs 0.0083 ± 0.0082; *p* > = 0.1), Latescibacterota (0.0005 ± 0.0011 vs 0.0002 ± 0.0008; *p* < 0.1), Myxococcota (0.0016 ± 0.002 vs 0.0005 ± 0.0011; *p* < 0.001), Nitrospirota (0.0037 ± 0.0031 vs 0.0024 ± 0.0046; *p* < 0.1), Patescibacteria (0.0028 ± 0.0025 vs 0.0025 ± 0.0024; *p* > = 0.1), Proteobacteria (0.1827 ± 0.0978 vs 0.1519 ± 0.0855; *p* > = 0.1), and Synergistota (0.0014 ± 0.0014 vs 0.0006 ± 0.0017; *p* < 0.01), although, Actinobacteriota (0.0258 ± 0.0147 vs 0.0571 ± 0.0634; *p* < 0.001), Cyanobacteria (0.0053 ± 0.0070 vs 0.0059 ± 0.0066; *p* > = 0.1), Deferribacterota (0.0056 ± 0.0054 vs 0.0066 ± 0.0057; *p* > = 0.1), Firmicutes (0.3752 ± 0.0491 vs 0.4265 ± 0.0689; *p* < 0.001), Gemmatimonadota (0.0004 ± 0.0006 vs 0.0005 ± 0.0013), Thermotogota (0.0002 ± 0.000319 vs 0.0003 ± 0.0008; *p* > = 0.1), Verrucomicrobiota (0.0052 ± 0.0042 vs 0.0053 ± 0.0045; *p* > = 0.1) phyla were lower in acute coronary syndrome group than healthy (**Figure 3C**).

Compared to the healthy group*, Lactobacillus* was observed higher (16% vs 13%) while *Bacteroides* (15.1% vs 21%) and *Lachnospiraceae_NK4A136_group* (16% vs 18%) were decreased in the ACS group compared to healthy. We further observed a significant higher proportion of *Brevibacillus* (0.0010 ± 0.0010 vs 0.000319 ± 0.000871; *p* < 0.001), *Brevibacterium* (0.0036 ± 0.0025 vs 0.001624 ± 0.0024; *p* < 0.001), *Candidatus_Solibacter* (0.0031 ± 0.0031 vs 0.0009 ± 0.0019; *p* < 0.001), *Chujaibacter* (0.0017 ± 0.0015 vs 0.000453 ± 0.000917; *p* < 0.001), *Kosakonia* (0.0071 ± 0.0048 vs 0.0032 ± 0.004401; *p* < 0.001), *Lachnospira* (0.0008 ± 0.0017 vs 0.000406 ± 0.000875; *p* > = 0.1), *Pantoea* (0.0061 ± 0.0040 vs 0.0037 ± 0.0043; *p* < 0.001), Parvimonas (0.0010 ± 0.0009 vs 0.00033 ± 0.00078; *p* < 0.001), *Phenylobacterium* (0.0012 ± 0.0015 vs 0.0004 ± 0.0011; *p* < 0.001) while, *Clostridium_sensu_stricto_1* (0.0036 ± 0.0057 vs 0.009147 ± 0.010172; *p* < 0.001), *Paenibacillus* (0.000086 ± 0.000358 vs 0.001591 ± 0.002693; *p* < 0.001), *Staphylococcus* (0.0217 ± 0.014 vs 0.0602 ± 0.0608; *p* < 0.001), were found decreased in acute coronary syndrome than healthy group (**Figure 3D**).

### Chronic coronary syndrome vs. healthy

Firmicutes and Bacteroidota (44% vs 43% and 15% vs 14%) were observed slightly increased while Proteobacteria (14% vs 15%) was found to decrease in the chronic coronary syndrome group than the healthy. Furthermore, the significant increased level of Cyanobacteria (0.0134 ± 0.0136 vs 0.0059 ± 0.0066; *p* < 0.001), Cloacimonadota (0.0002 ± 0.0009 vs 0.0000 ± 0.0002; *p* > = 0.1), Deferribacterota (0.008 ± 0.005 vs 0.006 ± 0.005; *p* < 0.1), Fusobacteriota (0.005 ± 0.005 vs 0.008 ± 0.008; *p* < 0.05), Desulfobacterota (0.014 ± 0.006 vs 0.0119 ± 0.0053; *p* < 0.01), Firmicutes (0.435 ± 0.070 vs 0.4265 ± 0.0689; *p* > = 0.1), Gemmatimonadota (0.0008 ± 0.0020 vs 0.0005 ± 0.0013), Myxococcota (0.0010 ± 0.0017 vs 0.0005 ± 0.0011; *p* > = 0.1), Patescibacteria (0.0042 ± 0.0034 vs 0.0025 ± 0.0024; *p* > = 0.1), Verrucomicrobiota (0.0075 ± 0.0071 vs 0.0053 ± 0.0045; *p* < 0.05) whereas, Acidobacteriota (0.005 ± 0.009 vs 0.0100 ± 0.0165; *p* > = 0.1), Actinobacteriota (0.021 ± 0.015 vs 0.0571 ± 0.063; *p* < 0.001), Latescibacterota (0.00006 ± 0.00043 vs 0.0002 ± 0.0008; *p* > = 0.1), Fusobacteriota (0.005 ± 0.005 vs 0.008 ± 0.008; *p* < 0.05), Nitrospirota (0.0015 ± 0.0027 vs 0.0024 ± 0.0046; *p* > = 0.1), Proteobacteria (0.1422 ± 0.0911 vs 0.1519 ± 0.0855; *p* > = 0.1), Synergistota (0.0003 ± 0.0009 vs 0.0006 ± 0.0017; *p* > = 0.1), and Thermotogota (0.00001 ± 0.00008 vs 0.0003 ± 0.0008; *p* < 0.01) were found decreased in chronic coronary syndrome group compare to healthy (**Figure 3C**).

A higher proportion of genus *Lachnospiraceae_NK4A136_group* (19.3% vs 17.9%) and *Lactobacillus* 24% vs 13%). Additionally, the significantly higher abundance of *Chujaibacter* (0.0003 ± 0.0012 vs 0.000453 ± 0.000917; *p* > = 0.1), *Lachnospira* (0.0030 ± 0.0045 vs 0.000406 ± 0.000875; *p* < 0.001), although a lower proportion of *Brevibacillus* (0.0000 ± 0.0003 vs 0.000319 ± 0.000871; *p* > = 0.1), *Brevibacterium* (0.0007 ± 0.0016 vs 0.001624 ± 0.0024; *p* < 0.05), *Candidatus_Solibacter* (0.0004 ± 0.0015 vs 0.000963 ± 0.001935 vs; *p* > = 0.1), *Clostridium_sensu_stricto_1* (0.0019 ± 0.0026; *p* < 0.001 vs 0.009147 ± 0.010172), *Kosakonia* (0.0023 ± 0.0036 vs 0.0032 ± 0.004401; *p* > = 0.1), *Paenibacillus* (0.000086 ± 0.000358 vs 0.001591 ± 0.002693; *p* > = < 0.001), *Pantoea* (0.0016 ± 0.0026 vs 0.0037 ± 0.0043; *p* < 0.01), *Parvimonas* (0.0010 ± 0.0009 vs 0.00033 ± 0.00078; *p* < 0.1), *Phenylobacterium* (0.0001 ± 0.0005 vs 0.0004 ± 0.0011; *p* > = 0.1), and *Staphylococcus* (0.0136 ± 0.013 vs 0.0602 ± 0.0608; *p* < 0.01) were observed in chronic coronary syndrome group compared to healthy (**Figure 3D**).

### Distinct blood microbiota in three groups

**Figure 4:**
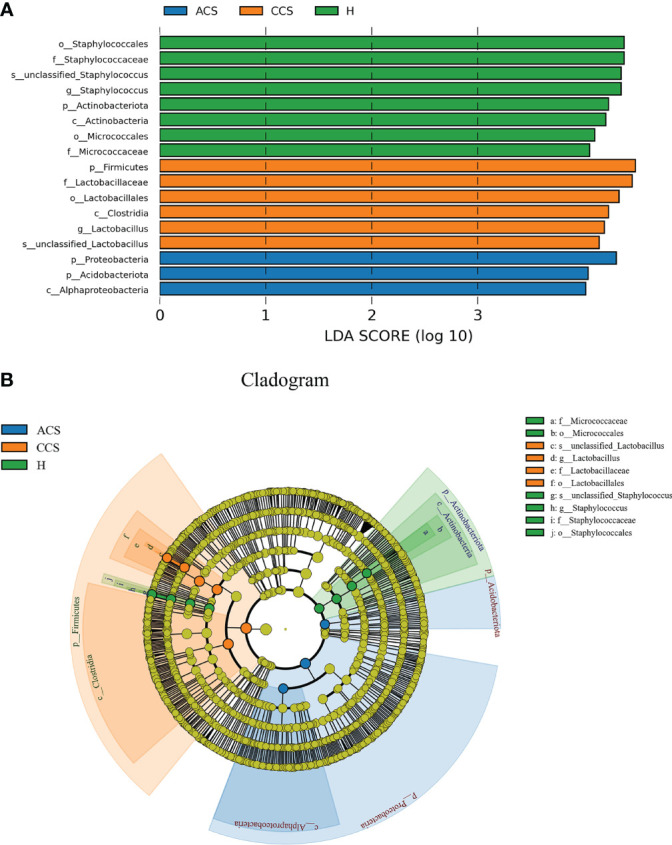
**(A)** LEfSe identified the most differentially abundant clades at all taxonomic levels among acute coronary syndrome, chronic coronary syndrome, and healthy groups using the LDA score of 3. **(B)** Cladogram of differentially abundant bacterial taxa wherein each layer represents a different taxonomy. The enriched taxa in acute coronary syndrome, chronic coronary syndrome, and healthy groups microbiota are represented in the cladogram. The central point represents the root of the tree (bacteria), and each ring represents the next lower taxonomic level (phylum to genus: p, phylum; c, class; o, order; f, family; g, genus).

As illustrated in (**Figure 4**), LEfSe modeling was used to confirm both the statistical and taxonomic differences between the blood microbiota of patients with acute coronary syndrome or chronic coronary syndrome and those of healthy, using a logarithmic LDA score cutoff of 3. LEfSe revealed substantial variations at several taxonomic levels across the three groups with a threshold score of LDA >3 after analyzing each participant’s taxonomic profile. Meanwhile, using linear discriminant analysis, the seventeen taxa were found significantly distinct among the three groups. Of them, phylum_ Actinobacteria, class_ Actinobacteria, order_ Staphylococcales, and Micrococcales, family_ Staphylococcaceae and Micrococcaceae, genus_ *Staphylococcus*, and Species_ Unclassified *Staphylococcus* in healthy group; phylum_ Firmicutes, class_ Clostridia, order_ Lactobacillales, family_ Lactobacillaceae, genus_ *Lactobacillus*, and Species_ Unclassified *Lactobacillus* in chronic coronary syndrome group, and phyla_ Proteobacteria and Acidobacteriota, class_ Alphaproteobacteria in acute coronary syndrome group were significantly different among three groups (**Figure 4A**). Additionally, (**Figure 4B**) shows the microbiome differences between the three groups at various phylogenic levels using a cladogram.

### COG annotation and analysis

The functional analysis through COG analysis among three groups showed that significantly up-regulated pathways such as energy production and conversion, intracellular trafficking, secretion, and vesicular transport, coenzymes transport and metabolism, lipid transport and metabolism, cell motility, secondary metabolites biosynthesis, transport and catabolism, posttranslational modification. Protein turnover, chaperones, chromatin structure and dynamics, amino acid transport, and metabolism were observed in the acute coronary syndrome group compared to the chronic coronary syndrome group. Although nucleotide transport and metabolism, replication, recombination and repair, cell cycle control, cell division, chromosome partitioning, translation, ribosomal structure and biogenesis, carbohydrates transport and metabolism, and transcription were found significantly up-regulated in chronic coronary syndrome patients than acute coronary syndrome group (**Supplementary Figure S4**).

In addition, a comparison between the acute coronary syndrome group with Healthy showed intracellular trafficking, secretion, and vesicular transport, cell motility, cell wall, membrane, envelope biogenesis, extracellular structures, posttranslational modification. Protein turnover, chaperones, cytoskeleton, and signal transduction mechanisms pathways were considerably up-regulated in the acute coronary syndrome group than healthy, while carbohydrates transport and metabolism, inorganic ion transport and metabolism, and transcription pathways were significantly up-regulated in the healthy group compared to acute coronary syndrome (**Supplementary Figure S5**).

Furthermore, the chronic coronary syndrome and healthy group comparison showed that the COG functional pathways such as, Cell cycle control, cell division, chromosome partitioning, Nucleotide transport, and metabolism, replication, recombination, and repair, translation, ribosomal structure and biogenesis, cell wall, membrane, envelope biogenesis, intracellular trafficking, secretion vesicular transport, and cytoskeleton were significantly up-regulated in chronic coronary syndrome group than healthy, whereas, energy production and conversion, coenzymes transport and metabolism, amino acid transport and metabolism, inorganic ion transport and metabolism, general function prediction only, lipid transport and metabolism, RNA processing and modification functional pathways were significantly up-regulated in the healthy group compared to chronic coronary syndrome. The significance was judged by a *p*-value less than *p* < 0.05 (**Supplementary Figure S6**).

### KEGG function annotation and analysis

KEGG-based functional annotations between acute coronary syndrome and chronic coronary syndrome analysis showed that the majority of pathways were significantly up-regulated in the acute coronary syndrome group than chronic coronary syndrome such as Amino acid metabolism, substance dependence, energy metabolism, global and overview maps, lipids metabolism, metabolism of cofactors and vitamins, circulatory system, sensory system, cell growth and death, aging, xenobiotic degradation and metabolism, neurodegenerative diseases, cancers; specific types, and drug resistance: antineoplastics. Although infectious diseases: bacterial, immune diseases, nucleotide metabolism, transcription, carbohydrate metabolism, replication and repair, membrane transport, drug resistance: antimicrobial, translation, signaling molecules and interaction, folding, sorting and degradation, and nervous system pathways were considerably upregulated in chronic coronary syndrome group compared to acute coronary syndrome (**Supplementary Figure S7**).

Additionally, acute coronary syndrome and healthy groups revealed that the majority of gene pathways were significantly up-regulated in the acute coronary syndrome group compared to the healthy such as cell growth and death, circulatory system, digestive system, cardiovascular diseases, drug resistance: antineoplastics, cancers; specific types, endocrine and metabolic diseases, sensory system, infectious diseases; viral, cell motility, signal transduction, drug resistance: antimicrobial, and cancer: overview genes, while global and overview maps, immune diseases, membrane transport, carbohydrate metabolism pathways were up-regulated in the healthy group compared to acute coronary syndrome (**Supplementary Figure S8**).

Moreover, comparing chronic coronary syndrome and healthy groups showed that the KEGG functional genes pathways such as infectious diseases: bacterial, drug resistance: antimicrobial, transcription, nucleotide metabolism, endocrine, and metabolic diseases, translation, signaling molecule and interaction, cell growth and death, replication, and repair, folding, sorting and degradation, cancer: overview, digestive system, biosynthesis of other secondary metabolites, cellular community-prokaryotes, and glycan biosynthesis and metabolism were significantly up-regulated in chronic coronary syndrome group compare to healthy, although global and overview maps, amino acid metabolism, metabolism of cofactor and vitamins, aging, lipid metabolism, substance dependence, transport, and catabolism pathways were observe significantly up-regulated in the healthy group than chronic coronary syndrome. The significance was judged by a *p*-value less than 0.05 (**Supplementary Figure S9**).

### Correlation between clinical parameters and microbial taxa

**Figure 5:**
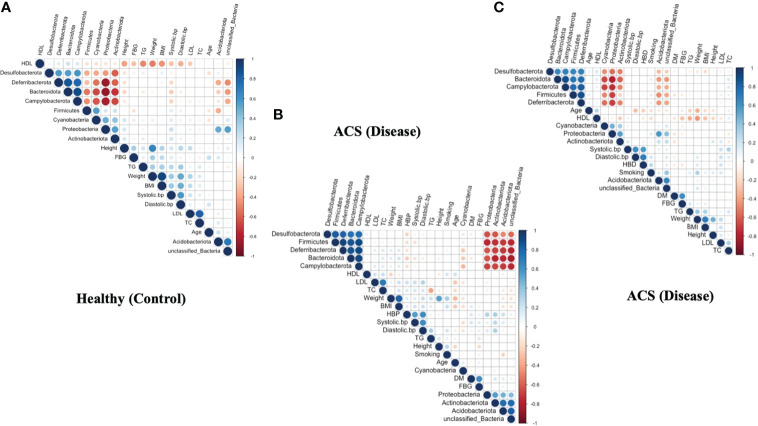
Correlation between clinical parameters and microbial taxa at the phylum level. **(A)** shows a correlation between clinical parameters and microbial taxa in the ACS group. **(B)** indicates a correlation between clinical parameters and microbial taxa in the CCS group. **(C)** indicates a correlation between clinical parameters and microbial taxa in the Healthy group.

To demonstrate pairwise comparisons of clinical variables, a color gradient denoting Spearman’s correlation coefficient was used (**Figure 5**). Clinical considerations were primarily focused on acute coronary syndrome and chronic coronary syndrome prognostic risk factors. Phylum-level correlations between clinical parameters and healthy, acute coronary syndrome, and chronic coronary syndrome taxa were used to investigate the effect of clinical parameters on circulating microbial composition, as depicted in (**Figures 5A–C**). Herein, the current findings revealed that there was no significant influence of clinical parameters on bacterial diversity. Hence, it was hypothesized that acute coronary syndrome and chronic coronary syndrome may have a major impact on the bacterial composition of the blood.

## Discussion

Advances in high-throughput sequencing, as well as the development of a targeted metagenomics technique, can now be used to quantify and define the taxonomic profile of the microbiome present in tissues, particularly blood. Hence, the blood microbiome of patients with acute coronary syndrome, chronic coronary syndrome, and healthy controls was characterized in this study. We observed a significant variation in Alpha and beta diversity in the blood microbial community composition among all groups. Regarding the unavoidable contamination, we focused more on the differences in blood microbiota, which assists in the identification of disease-related changes. Hence, our negative control results revealed that microbiota in the blood is not due to the reagent contamination. To the best of our knowledge, no comprehensive analysis of the microbiome in the blood of patients with Acute and Chronic coronary syndrome has been previously performed in China. To bridge that gap, we used Illumina NovaSeq to analyze the blood of Acute and Chronic Coronary Syndrome patients as well as healthy individuals.

The circulating microbiome patterns of patients with acute and chronic syndromes and healthy controls were shown to be significantly clustered. Differential abundance of various phyla and genera of bacteria could explain the differences, indicating a shift in the circulating microbiome in individuals with acute and chronic syndrome. The bacterial phyla Firmicutes, Bacteroidetes, Proteobacteria, and Actinobacteria dominate the blood collected from patients and controls. This result is somewhat similar to the findings reported by previous studies (McLaughlin et al., 2002; Amar et al., 2011; Dinakaran et al., 2014; Païssé et al., 2016; Gosiewski and Huminska, 2017; Li et al., 2018; Olde Loohuis et al., 2018; Qiu et al., 2019; Whittle et al., 2019). Proteobacteria, followed by Actinobacteria, Cyanobacteria, and Verrucomicrobia, were found to be responsible for cardiovascular disease in a prior study (Huse et al., 2008; Hillman et al., 2017). In this regard, we discovered that the Proteobacteria phylum was prominent in acute coronary patients; nevertheless, little is known about the role of changes in the microbiome’s quality and composition in cardiovascular disease. A landmark cohort study, the study examined the long-term relationship between circulating microbial fingerprints and cardiovascular events in the general population, independent of traditional cardiovascular risk factors, larger (vs. lower) relative abundance of the Proteobacteria phylum in peripheral blood leukocytes was related to a higher risk of incident cardiovascular events (Amar et al., 2013). According to a second cross-sectional study, patients with cardiovascular disease had a substantially higher relative abundance of the Proteobacteria phylum in their total blood than participants who appeared to be healthy (Rajendhran et al., 2013), which was similar to this finding. As a result, alterations in the human blood microbiome have been proposed as a “marker” for cardiovascular disease prediction. Increased populations of Proteobacteria in the gut and blood have been associated with inflammatory bowel disease, metabolic syndrome, cardiovascular disease, chronic lung disease, and atherosclerotic plaques (Amar et al., 2013; Calandrini et al., 2014; Rizzatti et al., 2017).

Proteobacteria also produce Lipopolysaccharides (LPS), which have been discovered to be a substantial component (Amar et al., 2011), several studies have reported on the function of LPS in the genesis of atherosclerosis (Carnevale et al., 2020; Hashimoto et al., 2020; Violi et al., 2020; Zhou et al., 2020). Our data show a negative correlation between bacterial DNA levels and baseline fasting blood glucose levels. As demonstrated in a healthy human model of gram-negative sepsis, we can’t rule out a transient improvement in insulin sensitivity in the early stages of metabolic infection (Van Der Crabben et al., 2009). This could be because the latent infection causes the release of nitric oxide, which improves insulin sensitivity (Perreault and Marette, 2001). In the blood of the acute coronary syndrome group, we found a significant amount of Proteobacteria phylum. As a result, we conclude that circulating microorganisms may be the primary source of microbial colonization of atherosclerotic plaques, leading to inflammation and cardiovascular disease.

A significantly increased level of Desulfobacterota was found in the acute coronary syndrome although its proportion was considerably decreased in the chronic coronary syndrome group. Desulfobacterota is a phylum of bacteria that uses the DsrAB-dissimilatory sulfate reduction pathway to reduce sulfur compounds (Wang et al., 2019), Desulfobacterota also participates in butyrate breakdown *via* the butyrate beta-oxidation pathway, indicating that they are involved in the catabolic reaction’s equilibrium (Hao et al., 2020). As a result, bacteria belonging to the Bacteroidetes and Desulfobacterota phyla may release LPS, causing inflammatory damage or aggravating energy metabolism irregularities, both of which are pathological features of diabetes and linked to an increased risk of cardiovascular disease. Herein, in the chronic coronary syndrome group, Desulfobacterota were significantly increased although the proportion of Proteobacteria was significantly decreased which is oblivious different from the chronic coronary syndrome group. However, the direct role of Desulfobacterota in atherosclerosis is not yet been confirmed.

At the genus level, compared to that in the control, the increased level of pathogenic genera *Brevibacterium, Kosakonia, and Lactobacillus* were detected in the blood of the present cohort, *Brevibacterium*, and *Kosakonia* were previously detected in the bloodstream infections (Bhatti et al., 2017; Lelouvier et al., 2017; Asai et al., 2019). *Brevibacteria* is gram-positive obligate aerobic rods that relate to milk and can also be detected on human skin. *Brevibacterium* has been identified as an uncommon cause of catheter-related bloodstream infection, primarily in immunocompromised patients with cancer or AIDS (Asai et al., 2019). Based on previous study, Brevibacteria is a rare but important organism that can cause opportunistic infections in immunocompetent people. Bacterial translocation induces systemic inflammation, which contributes to MI comorbidities and heart failure, as well as the risk of bloodstream infection through different routes in patients with MI (Zabell and Tang, 2017).

*Lactobacillus* is rarely a human pathogen but has also been reported to cause dental caries (Ahirwar et al., 2019), infective endocarditis (Husni et al., 1997), urinary tract infections (Reid et al., 1992; Grin et al., 2013), chorioamnionitis (Martius and Eschenbach, 1990), endometritis (Wang et al., 2018), meningitis (Cannon et al., 2005), intraabdominal abscess (Husni et al., 1997), liver abscess (Pararajasingam and Uwagwu, 2017), splenic abscess (Sherman et al., 1987), and bacteremia (Antony et al., 1996; Husni et al., 1997). *Lactobacillus* abundances were shown to be higher in these STEMI patients, probably due to abnormalities in the gut barrier’s tight junctions. (Zhou et al., 2018). Furthermore, a significantly high abundance of *Lactobacillus* was found in the gut of coronary artery disease patients (Zhu et al., 2018). Changes in circulating microorganisms could lead to chronic infection and inflammatory reactions, which could lead to cardiovascular diseases.

Our study also found a slight increase level of *Escherichia-Shigella* which is recently found in several diseases. A study demonstrated the higher abundance of *Escherichia-Shigella* in coronary artery disease patients (Zhu et al., 2018), Another study discovered numerous bacteria in the hepatic vein blood that were linked to the levels of inflammatory cytokines. The strongest relationships with IL-8 were found in *Escherichia coli* and *Prevotella*. This could be due to these bacteria’s propensity to produce LPS and cause inflammation *via* TLR or inflammasome cascades (Gedgaudas et al., 2022). Proteobacteria also produce LPS which has a direct role in cardiovascular diseases, it may suggest that phylum Proteobacteria and genus *Escherichia-Shigella* might play a potential role in chronic coronary syndrome. However, the direct role of *Escherichia-Shigella* in the chronic coronary syndrome is still not clear

Moreover, we measured alpha and beta diversity, Alpha diversity measures the richness of bacterial taxa within each sample (Wallenborn et al., 2021). Overall, the acute coronary syndrome group had significantly higher alpha diversity indices (Shannon and Chao1) than the chronic coronary syndrome and healthy groups, while the chronic coronary syndrome group had significantly lower alpha diversity than the healthy group. Recently higher alpha diversity was observed in non-communicable diseases (Vujkovic-Cvijin et al., 2020). Also, higher alpha diversity was found in the tumor microbiome of long-term survival patients (Riquelme et al., 2019). Additionally, the microbiome-based Hippurate prediction score controlled the clinical relationship pattern of microbial diversity, suggesting that benzoate metabolism may play a role in the beneficial connections between high alpha diversity and good health (Hertel et al., 2022). Even though chronic coronary syndrome groups had much lesser alpha diversity, a dysbiotic gut has been linked to autoimmune diseases, diabetes, metabolic syndrome, autism, and colorectal cancer, among other chronic disorders (Mosca et al., 2016). Vascular stiffness and (Menni et al., 2018), type 2 diabetes (Chakaroun et al., 2021), have also been linked to a decrease in alpha diversity. According to recent research, chronic renal disease patients’ blood has less alpha diversity (Shah et al., 2019), than patients with myocardial infarction (Amar et al., 2019). Our chronic coronary syndrome group’s findings are comparable with those of prior studies, implying a possible link between sickness and decreased bacterial diversity.

Beta diversity is used to calculate and compare variation between three groups (Lozupone et al., 2007). The blood bacterial composition of patients with acute coronary syndrome, chronic coronary syndrome, and healthy showed moderate differences in beta diversity (PCoA analysis). A similar outcome was discovered by (Kamo et al., 2017), in heart failure patients and healthy participants of the gut microbiota (Mayerhofer et al., 2020). Significantly distinct beta diversity of the gut oral, thrombus has also been observed in Korean individuals with ST-elevation myocardial infarction (Kwun et al., 2020). This study’s findings demonstrated a partial split between the two groups. Together, these findings suggest that beta diversity may play a role in both acute and chronic coronary syndromes. As a result, we believe that changes in the blood microbiota have a role in the initiation and progression of atherosclerosis. In addition, a study evaluating the correlations between clinical measures and microbial taxa in the acute coronary syndrome and chronic coronary syndrome groups found no significant link between blood microbiota composition and clinical variables. Our previous study is supported by these findings (Khan et al., 2022a).

This study also investigated the characteristics and potential activities of blood microbiota in acute coronary syndrome and chronic coronary syndrome patients, revealing new insights into the etiology of atherosclerosis and identifying prospective bacterial biomarkers. In atherosclerosis patients, COG and KEGG pathway analyses revealed a lower abundance of some key metabolic and transport pathways. Blood microbiota dysbiosis may play a role in atherosclerosis-related host metabolic diseases. The findings could lead to a new understanding of atherosclerosis pathophysiology and the development of novel treatment alternatives in the future. Circulating microbiota-related metabolites could be useful in preventing atherosclerosis in these circumstances.

### Strengths and limitations of the study

Considering multiple recent studies that showed changes in blood microbiota composition and diversity are linked to non-communicable diseases, by examining changes in blood microbial signature among acute coronary syndrome, chronic coronary syndrome, and Healthy group, we hope to determine the role of circulating microbiota on atherosclerosis. In addition, our results revealed that the patients with acute coronary syndrome and chronic coronary syndrome were significantly older than healthy individuals, these findings are similar to our previous results (Khan et al., 2022b), this suggests that another important factor influencing atherosclerosis is age, a detailed study worth needed to investigate the blood microbiota profile between young and aged individuals. Improving the accuracy of risk prediction is very important because it helps target those subjects who are most likely to benefit from preventive treatment.

Although the current study’s findings were substantial, the study’s main limitation is the small sample size, which may impair the accuracy of the findings due to rarity. A larger study cohort was needed to investigate the effects of blood microbiota signature. Meanwhile, more modern techniques such as metagenomics and metabonomics must be used to investigate the potential health effects of specific circulating microbiota and their metabolites.

## Conclusion

In conclusion, the current study found that atherosclerosis patients and healthy controls have distinct blood flora diversity and composition. A change in blood microbiota may be linked to the development of atherosclerosis. A causal relationship between atherosclerosis and blood flora requires more research.

## Data availability statement

The datasets presented in this study can be found in online repositories. The names of the repository/repositories and accession number(s) can be found in the article/**Supplementary Material**.

## Ethics statement

The studies involving human participants were reviewed and approved by Medical Ethics Committee of the Northwest Minzu University, Gansu, China. The patients/participants provided their written informed consent to participate in this study.

## Author contributions

AL conceived, initiated, and supervised the research; LZ, ZJ, and IkK designed the experiment and analyzed the resulting data; ZW and XP helped with sample collection and provided the participant’s data information; ImK and MU helped in compiled all Figures and statistical analysis and in the revised manuscript. IkK wrote and ran the informatics pipeline with the help of all authors. LZ and ZJ gave conceptual advice and revised the manuscript. All authors contributed to the article and approved the submitted version.

## Funding

The study was supported by the “National Natural Science Foundation of China” and the project approval number is 31760159.

## Acknowledgment

We thank the Gansu Provincial Hospital and Lanzhou University Second hospital for providing blood samples, and their health information data. In addition, we gratefully acknowledged all authors for their contribution and support.

## Conflict of interest

The authors declare that the research was conducted in the absence of any commercial or financial relationships that could be construed as a potential conflict of interest.

## Publisher’s note

All claims expressed in this article are solely those of the authors and do not necessarily represent those of their affiliated organizations, or those of the publisher, the editors and the reviewers. Any product that may be evaluated in this article, or claim that may be made by its manufacturer, is not guaranteed or endorsed by the publisher.
