# Supplementary material for: Analysis of the blood bacterial composition of patients with acute coronary syndrome and chronic coronary syndrome
Source: Front Cell Infect Microbiol. 2022 Oct 4;12:943808. doi: 10.3389/fcimb.2022.943808 (PMC9577097; doi:10.3389/fcimb.2022.943808)
Supplement: Supplementary file 1 [file DataSheet_1.pdf]

Table S1. Comparison of blood microbiota between acute chronic syndrome, chronic coronary syndrome, and healthy groups at Phylum level.

**Phylum**	**ACS vs CCS**	**ACS vs Healthy**	**CCS vs Healthy**
Firmicutes	39% vs 45%	39% vs 43%	44% vs 43%
Bacteroidota	31% vs 32%	31% vs 29%	32% vs 29%
Proteobacteria	19% vs 15%	19% vs 15%	14% vs 15%
Actinobacteria	3% vs 2%	3% vs 6%	2% vs 6%
Campylobacterota	1% vs 1%	1% vs 1%	1% vs 1%

The percentage comparison of the blood microbiome at the phyla level among the three groups is presented. Acute coronary syndrome group vs Chronic coronary syndrome (ACCS vs CCs); Acute coronary syndrome vs healthy (ACS vs Healthy); Chronic coronary syndrome vs healthy (CCS vs healthy).

Table S2. Composition of top ten high abundance genera in the blood of Acute coronary syndrome, chronic coronary syndrome, and healthy groups.

**Genus**	**ACS vs CCS**	**ACS vs Healthy**	**CCS vs Healthy**
Lachnospiraceae_NK4A136_group	16% vs 19%	16% vs 18%	19% vs 18%
Lactobacillus	16% vs 24%	16% vs 13%	24% vs 13%
Ligilactobacillus	10% vs 15%	10% vs 9%	15% vs 9%
Staphylococcus	8% vs 5%	8% vs 22%	5% vs 22%
Bacteroides	15% vs 9%	15% vs 10%	9% vs 10%
Escherichia_Shigella	8% vs 11%	8% vs 10%	11% vs 10%
Alloprevotella	8% vs 8%	8% vs 8%	8% vs 8%
Massilia	8% vs 1%	8% vs 2%	1% vs 2%
Bacillus	7% vs 3%	7% vs 4%	3% vs 4%
Helicobacter	5% vs 5%	5% vs 5%	5% vs 5%

Data are shown in percentage level. Acute coronary syndrome group vs Chronic coronary syndrome (ACCS vs CCs); Acute coronary syndrome vs healthy (ACS vs Healthy); Chronic coronary syndrome vs healthy (CCS vs healthy).

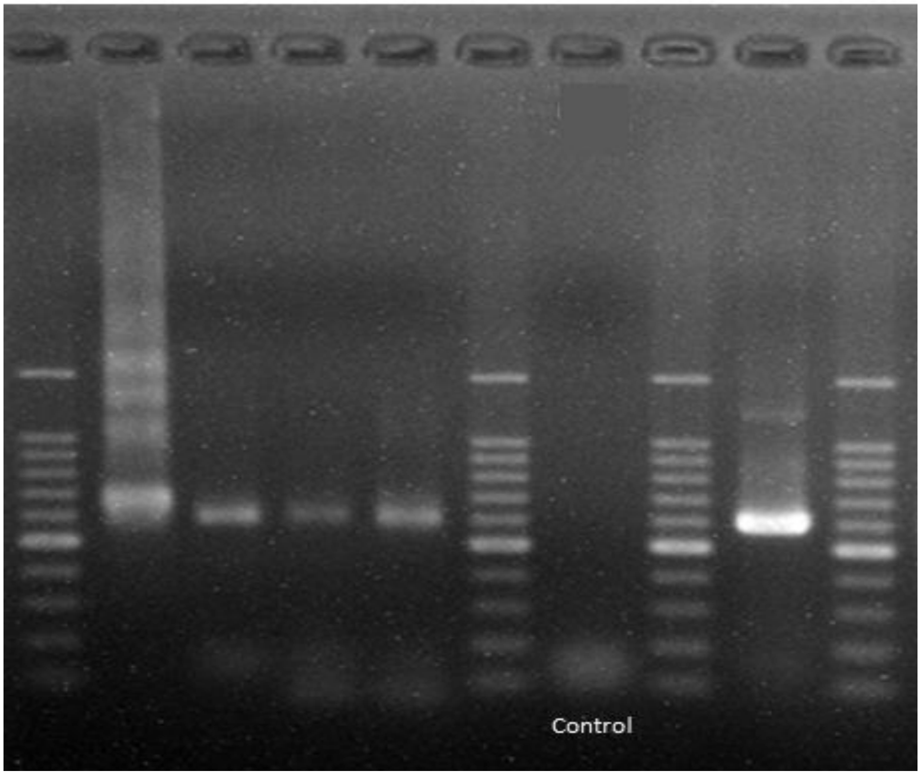

Figure S1 | The PCR results of the negative control samples demonstrate that the PCR experiment was not contaminated.

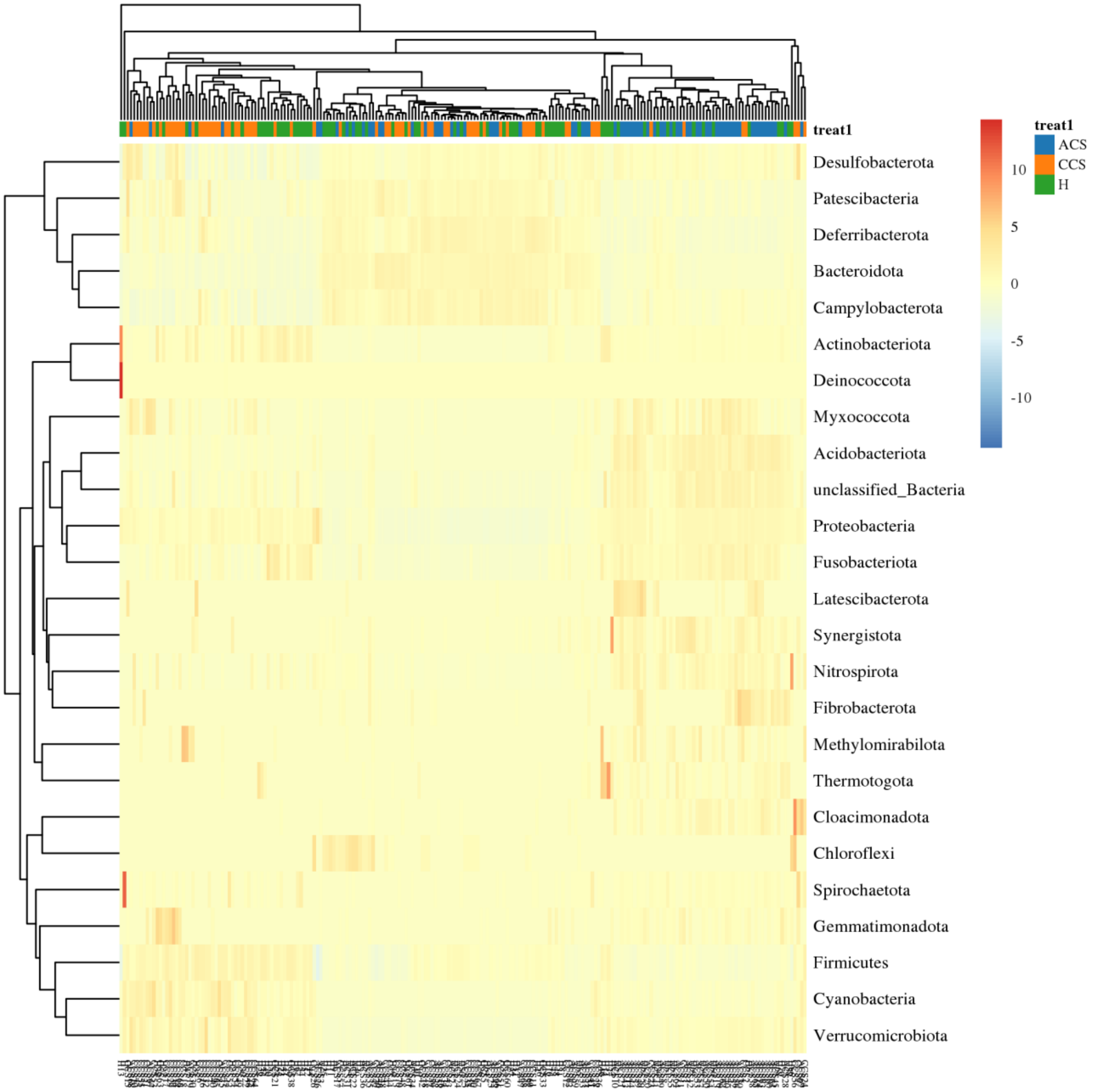

Figure S2| Heatmap represents the relative abundance of blood microbial taxa among three groups at the phylum level.

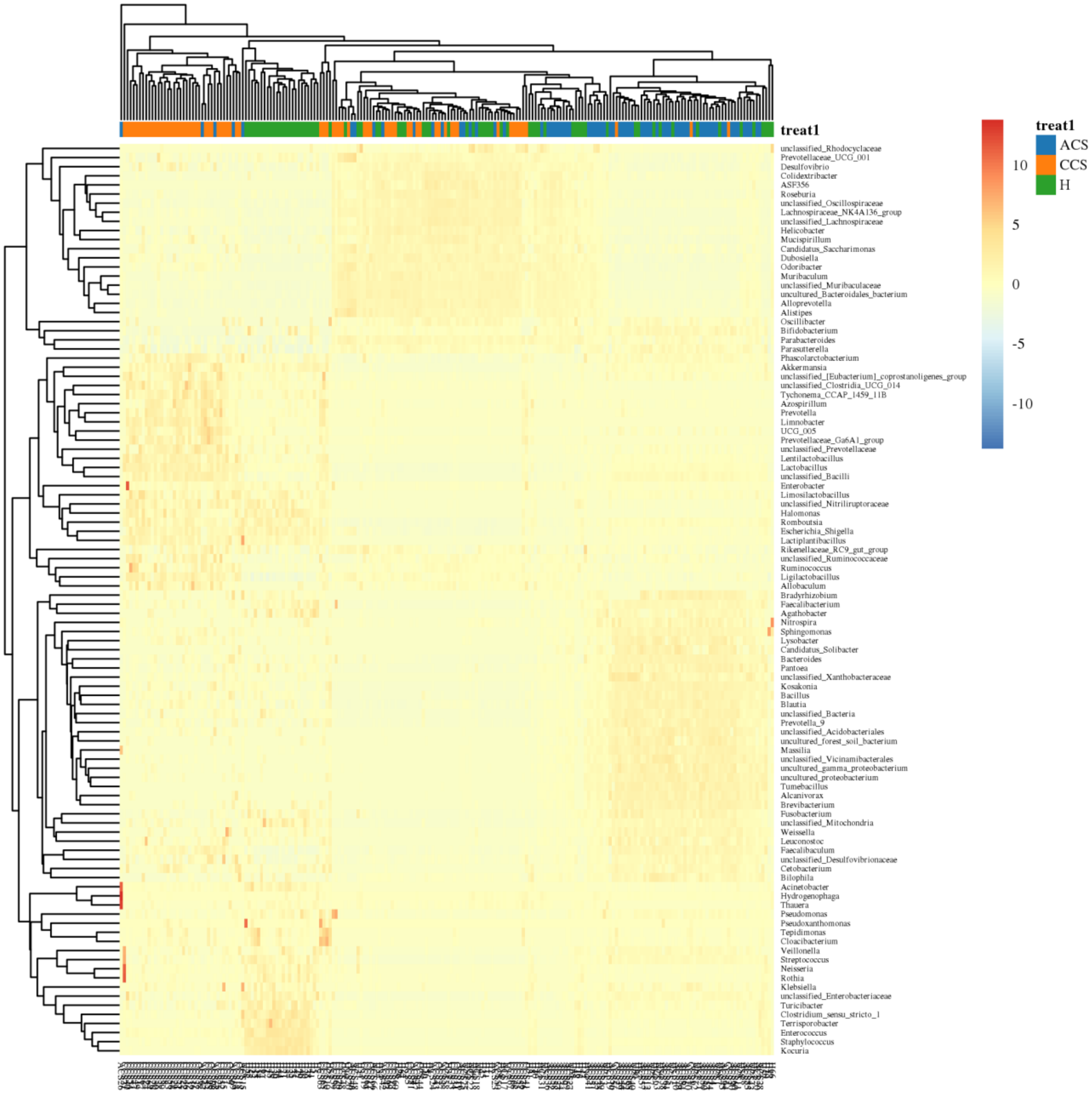

Figure S3 | The relative abundance of the blood microbial taxa among three groups at the genera level.

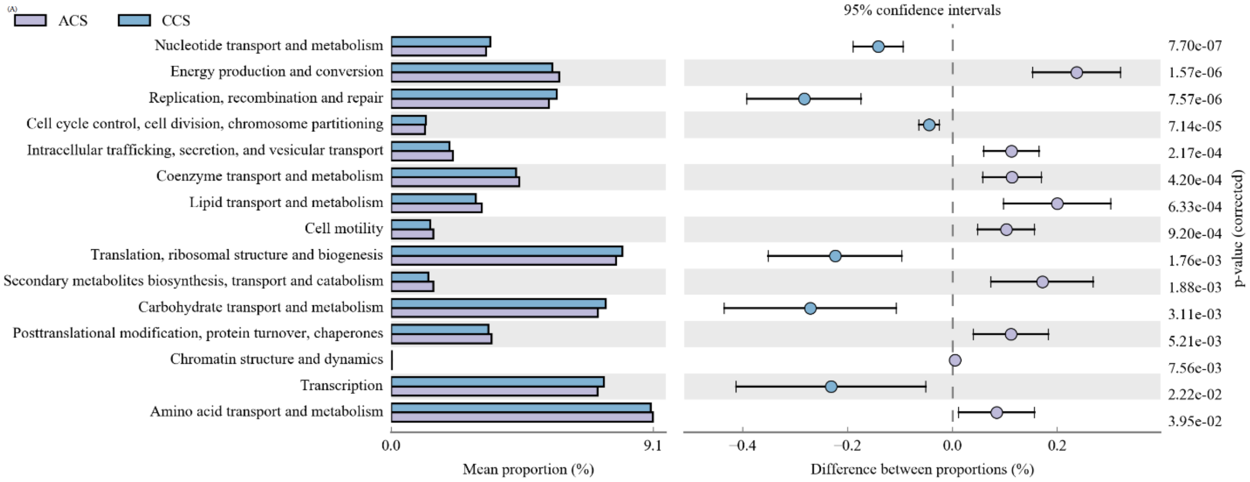

Figure S4 | Shows the Clusters of Orthologous Genes functional prediction analysis. (A) Distribution of predicted reads in the Clusters of Orthologous Genes classification between the Acute coronary syndrome and Chronic coronary syndrome groups.

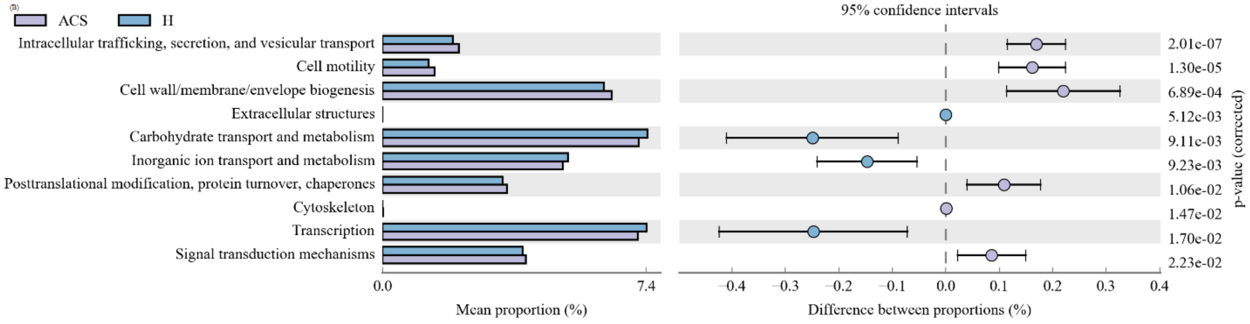

Figure S5 | Clusters of Orthologous Genes analysis reveals metabolic functions that are enriched or underrepresented in the blood microbiome between Acute coronary syndrome and the healthy group.

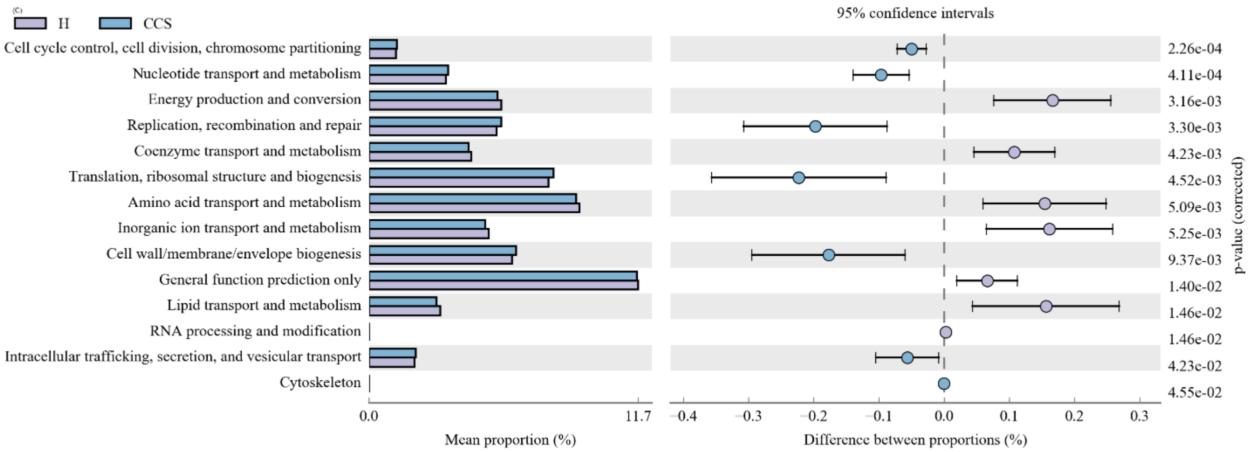

Figure S6 | The blood microbiota of the Chronic coronary syndrome and healthy groups demonstrates metabolic functions that are either enriched or underrepresented, based on the Clusters of Orthologous Genes assessment.

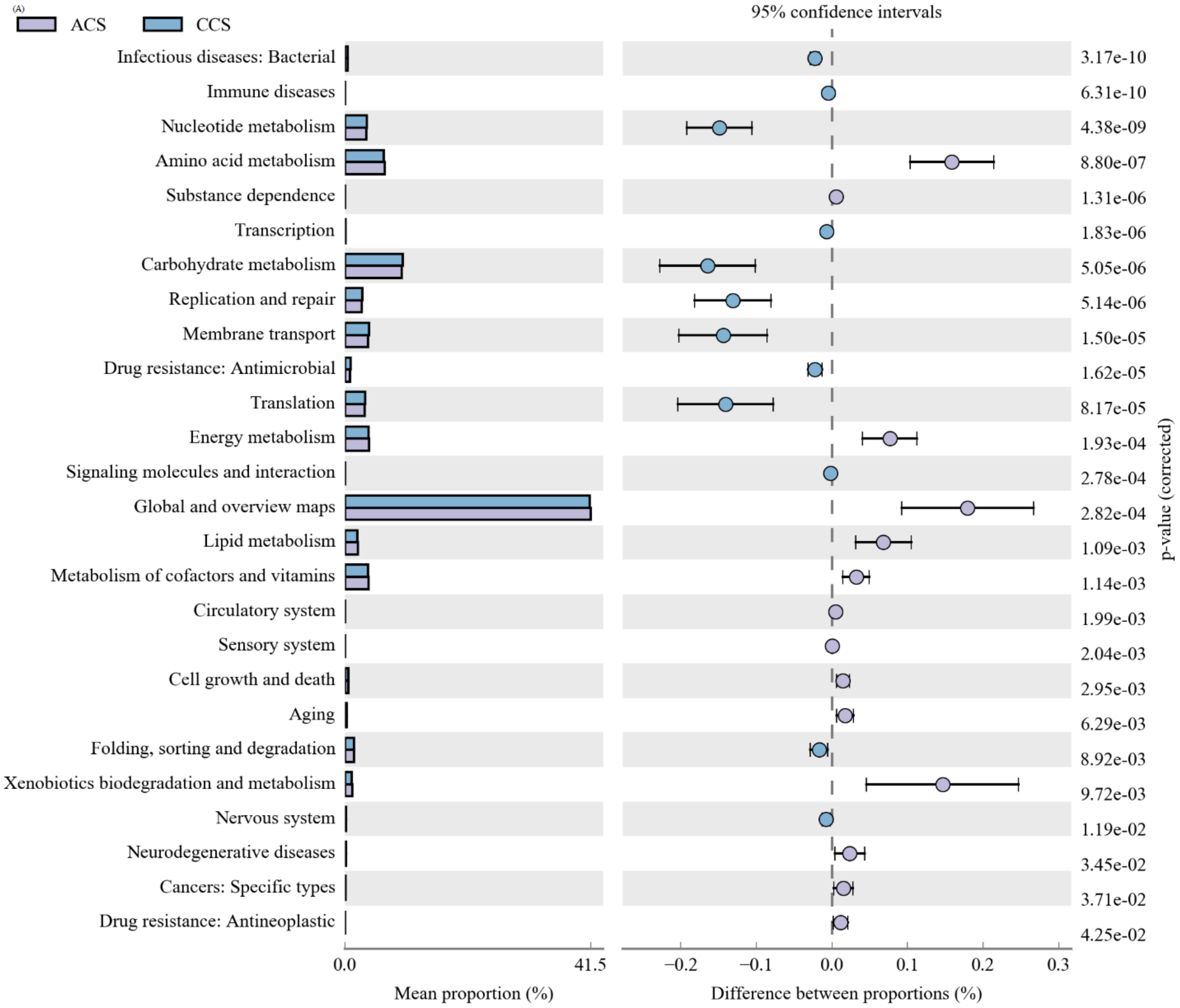

Figure S7 | Represent Kyoto Encyclopedia of Genes and Genomes functional prediction analysis. (A) indicates the expression of functional pathways between Acute coronary syndrome and Chronic coronary syndrome groups based on the Kyoto Encyclopedia of Genes and Genomes pathway.

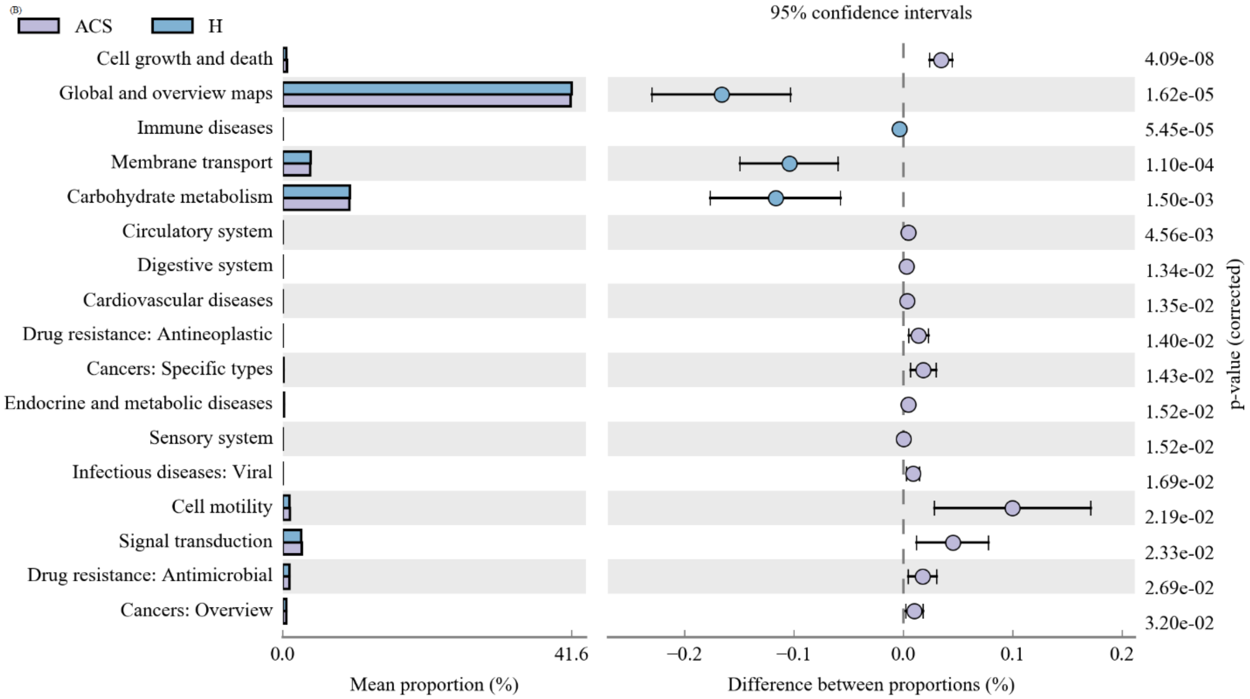

Figure S8 | Indicates the expression of functional pathways between Acute coronary syndrome and the healthy group.

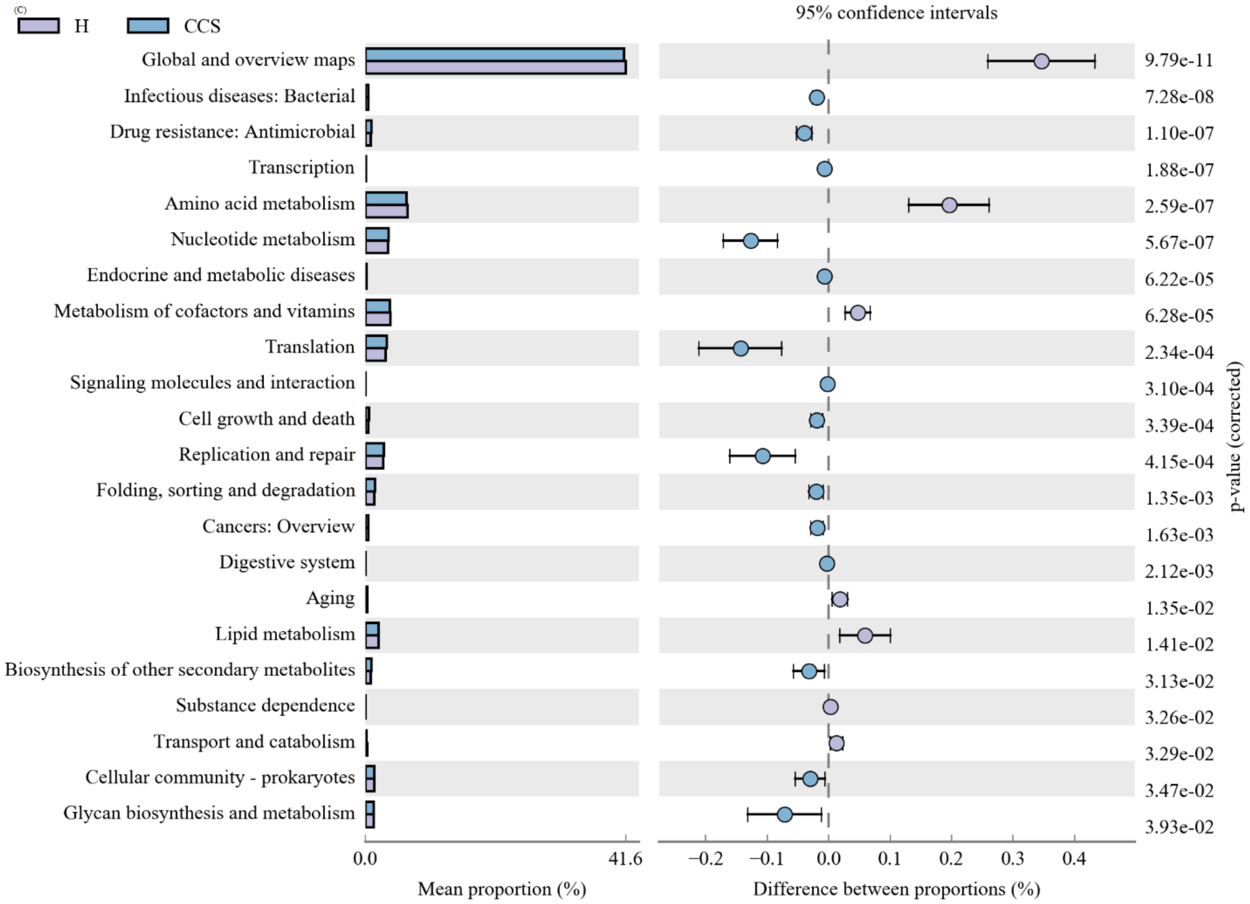

Figure S9 | Indicates the expression of functional pathways between Chronic coronary syndrome and healthy groups.
